# Detection of mind wandering using EEG: Within and across individuals

**DOI:** 10.1371/journal.pone.0251490

**Published:** 2021-05-12

**Authors:** Henry W. Dong, Caitlin Mills, Robert T. Knight, Julia W. Y. Kam

**Affiliations:** 1 Helen Wills Neuroscience Institute, University of California, Berkeley, Berkeley, California, United States of America; 2 Department of Psychology, University of New Hampshire, Durham, New Hampshire, United States of America; 3 Department of Psychology, University of Calgary, Calgary, Alberta, Canada; 4 Hotchkiss Brain Institute, Calgary, Alberta, Canada; Ulm University, GERMANY

## Abstract

Mind wandering is often characterized by attention oriented away from an external task towards our internal, self-generated thoughts. This universal phenomenon has been linked to numerous disruptive functional outcomes, including performance errors and negative affect. Despite its prevalence and impact, studies to date have yet to identify robust behavioral signatures, making unobtrusive, yet reliable detection of mind wandering a difficult but important task for future applications. Here we examined whether electrophysiological measures can be used in machine learning models to accurately predict mind wandering states. We recorded scalp EEG from participants as they performed an auditory target detection task and self-reported whether they were on task or mind wandering. We successfully classified attention states both within (person-dependent) and across (person-independent) individuals using event-related potential (ERP) measures. Non-linear and linear machine learning models detected mind wandering above-chance within subjects: support vector machine (AUC = 0.715) and logistic regression (AUC = 0.635). Importantly, these models also generalized across subjects: support vector machine (AUC = 0.613) and logistic regression (AUC = 0.609), suggesting we can reliably predict a given individual’s attention state based on ERP patterns observed in the group. This study is the first to demonstrate that machine learning models can generalize to “never-seen-before” individuals using electrophysiological measures, highlighting their potential for real-time prediction of covert attention states.

## Introduction

We frequently find ourselves drifting away from a conversation, our work, or the movie in front of us, towards our inner world. Often referred to as mind wandering, this phenomenon has been traditionally characterized as an attentional shift from externally oriented task-related thoughts to internally oriented task-unrelated thoughts [1–3]. More recently, the specific definition of mind wandering is under debate [4, 5]. Mind wandering has been associated with numerous benefits, including future planning, creativity, and problem solving [6–8]. However, previous studies have also established robust negative associations with mind wandering: including higher levels of negative affect [9, 10] and impaired performance in a variety of externally oriented tasks, such as target detection [11], performance monitoring [12], and reading comprehension [13–15]. Given the potential negative impact of mind wandering on performance, reliable detection of this phenomenon provides a step towards optimizing task performance in daily life.

Mind wandering is an inherently internal process that often occurs in the absence of overt behavioral markers, making it difficult to detect and combat in real-time. A unique barrier for mind wandering research is its current overreliance on self-reported experience. In particular, the most commonly used and well-validated approach to study mind wandering is the online thought sampling method [2, 16], in which subjects are occasionally interrupted throughout an externally oriented task and asked to indicate whether they were having a task-related thought (i.e. on task) or task-unrelated thought (i.e. mind wandering). One of the main advantages of thought sampling is that it provides a direct, in-the-moment measure of one’s attentional state. However, this approach may be impacted by demand characteristics or lack of awareness of attention state [16, 17]. Thought sampling may also change the nature of the task itself since it requires constant interruptions. Unobtrusive detection of mind wandering using machine learning methods thus offers a potential solution that overcomes these challenges and provides avenues for applications that can address the negative impacts of mind wandering in real-time [18]. Establishing the validation and effectiveness of machine learning in detecting mind wandering across contexts has the potential to eventually replace the need for thought sampling to determine the occurrence of mind wandering.

Previous successful attempts of mind wandering detection primarily used behavioral measures such as eye tracking and pupillometry [19–22] or task-related measures, such as driving performance [23, 24] and reading time [25]. Studies have also used physiological measures such as heart rate and skin conductance [26] as well as synchronization between respiration and sensory pressure [27]. These findings serve to highlight the value of using behavioral and physiological measures to detect mind wandering at above chance levels. Compared to these indirect markers, neural measures may be more effective at directly capturing this inherently covert attention state. Here, we assessed the utility of a neural measure for mind wandering detection by examining electrical activity originating from the brain using scalp EEG during this internally oriented state.

### Electrophysiological markers of mind wandering

Ample evidence from scalp EEG studies has established a distinct set of electrophysiological signatures of mind wandering, which is promising for real-time detection [28–34]. Given that scalp EEG is comparably low cost, and can be implemented outside of the laboratory, these EEG measures could be ideal for real-time detection of this phenomenon in the real world. In particular, the P1 and N1 ERP components in response to visual and auditory probes in a target detection task are reduced during mind wandering [33, 35, 36]. This indicates that sensory-evoked responses to both visual and auditory inputs were significantly attenuated, suggesting that mind wandering disrupts external perception regardless of sensory modality. Similarly, several studies have documented a reduction of the P3 ERP component during periods of mind wandering relative to on task [12, 34, 37, 38], demonstrating an overall attenuation of higher-level cognitive processes. Together, these findings suggest that mind wandering periods are associated with reduced external processing as observed in changes in ERP amplitude. These studies are consistent with the executive function model of mind wandering, which posits that in order to facilitate mind wandering, our executive resources are disengaged from the external task and instead directed internally to our thoughts [2, 7].

Prior EEG studies have also reported changes in low frequency power during mind wandering. Specifically, Braboszcz and Delorme reported increases in theta activity and decreases in alpha activity during mind wandering [28]. Using a similar experimental design, van Son and colleagues [39] found a higher ratio of theta and beta activity in the frontal cortex during mind wandering. In contrast to these findings, others have reported increased frontocentral theta power during external cognition [40–42], whereas increased posterior alpha power has been implicated in mind wandering [23, 38]. These variable patterns in low frequency power as a marker of mind wandering may be driven by differences in stimulus modality, experimental manipulation of top-down processes, or the electrode sites at which power was measured. Importantly, given the overlapping information between low frequency power and ERP components (typically measured between 1-30Hz), and that ERP patterns are relatively more consistent across studies, the current study used ERP measures as features in the machine learning models.

### Using EEG for mind wandering detection

Converging evidence points towards several reliable EEG correlates of mind wandering, and several studies to date have attempted to build detectors of mind wandering based solely on EEG measures. Kawashima and Kumano [43] used EEG signals (power and coherence in delta, theta, alpha, beta, and gamma frequency bands) to predict mind wandering intensity during a sustained attention to response task. They found that non-linear models using multiple electrodes resulted in higher prediction accuracy of mind wandering intensity than linear models using a single electrode. Jin and colleagues [44] extended this work by predicting mind wandering with a nonlinear model that generalized across tasks within individual subjects. Specifically, they used a support vector machine to predict mind wandering with EEG markers (including the P1, N1, and P3 ERP components, as well as theta and alpha power and coherence), reporting an average on task/off task classification accuracy of 60% that generalized across two visual tasks. This task generalization is noteworthy as it suggests that models trained on EEG markers may detect mind-wandering without needing to first train on new tasks. This group [45] also demonstrated that mind wandering is not dependent on and is quantitatively different from subject vigilance and current task demands, by reporting that a classifier trained through thought sampling outperformed classifiers trained through either vigilance or task demands. Dhindsa and colleagues [46] extended these findings by detecting mind wandering in real world settings. They recorded EEG activity during live lectures and used frequency band power measurements (theta, alpha, and beta) to achieve an average detection accuracy of 80–83%. Finally, Tasika and colleagues [47] employed a multi-step framework that leverages a J48 decision tree classifier and a support vector machine with a radial basis function kernel to detect mind wandering using EEG, and reported an average accuracy as high as 84.49% based on two individuals.

An important property that is not clearly addressed in previous work, however, is the generalizability of models across participants: a model that identifies an optimal set of features at the group level that can accurately predict attention states of another individual not in that group (i.e. person-independent). Although this approach typically results in overall lower classification performance, it allows for more flexible generalizability for real-time prediction. Previous attempts using machine learning and EEG measures exclusively to detect mind wandering used data from the same individuals to train and test the models. In other words, there was dependence within individuals, which means the models likely do not generalize well to new subjects. We aim to improve on mind wandering detection using only EEG by building models that generalize across individuals in a person-independent manner.

This proof-of-concept study examined whether machine learning models using EEG measures can detect mind wandering 1) *within* individuals and 2) *across* individuals. Using thought sampling during a target detection task, we asked subjects to report their attention state throughout the task as we recorded their EEG. Our study included EEG markers, specifically ERP components that have previously demonstrated reliable attentional differences, as features in two machine learning models. To our knowledge, this is the first study to establish that machine learning using EEG features is capable of detecting mind wandering both within individuals (i.e. person-dependent) and across individuals (i.e. person-independent).

To address this issue, we asked participants to perform an auditory target detection task while their EEG is being recorded. In order to obtain a measure of participants’ attention state in the moment, participants were asked to report their attentional state as on task or mind wandering at pseudorandom intervals throughout the task. We extracted EEG measures of interest (namely N1 and P3 ERP) as a function of the reported attention states. Using these EEG measures, we built linear and non-linear machine learning models to determine whether these measures can be used to classify attention states both within and across individuals, and if so, which models led to superior performance. For classification within subjects, we used each subject’s own EEG markers of attention state for prediction, which allowed us to maximize prediction accuracy for that individual. For classification across subjects, our models trained on one set of data and attempted to identify an optimal algorithm that can predict attentional state of individuals not part of the training group data. Together, these two approaches help determine whether machine learning with EEG measures can accurately predict mind wandering within and across individuals.

## Methods

### Subjects

Fourteen subjects participated in the experiment (9 females, 5 males; age range: 24–75, *M* ± *SD* = 51.9± 14.7). Although the sample size appears small, it is sufficient for addressing our primary aim of mind wandering detection. Specifically, it is comparable with previous studies using within-subject training and testing sets that included fewer than 20 subjects to detect mind wandering using EEG features (i.e. features that were derived from EEG data) [44, 46]. Further, if we can predict mind wandering with this sample size for the across-subject analyses, this finding provides a lower threshold necessary for accurately detecting mind wandering using EEG features. All subjects provided informed written consent and were reimbursed for their participation. This study was approved by the Institutional Review Board at the University of California, Berkeley.

### Task stimuli and paradigm

Subjects sat in a dark room and performed an auditory target detection task [36]. They were presented with a series of standard tones (800Hz) and target tones (1000Hz) in a random order with probabilities of 0.8 and 0.2, respectively. There was a total of 1500 tones, 1200 of which were standard tones and 300 of which were target tones. Each sound was a pure tone presented at 75 dB SL that lasted 200ms, and the inter-trial interval was randomly jittered between 800-1200ms. Subjects were instructed to press a button to target tones as quickly and accurately as possible. They were asked to keep their eyes fixated on the cross in the center of the screen at all times. Reaction time to the target tones was recorded, and accuracy was considered across both tones (i.e. detection of target tones and correct rejection of standard tones). Attentional differences in behavioral measures were assessed using dependent samples t-tests.

During the task, we occasionally presented thought probes that asked subjects to report their attention state as either “on task” or “mind wandering.” To ensure subjects understood the meaning of these attention states, we provided them with clear and detailed definitions and examples. “On task” was defined as one’s attention being firmly directed towards the target detection task, and “mind wandering” was defined as one’s attention being oriented away from the task. For each thought probe, we extracted the 10 trials (approximately 15 seconds) prior to the probe, and labeled these 10 trials according to the subject’s response to the thought probe. For instance, if the response to one thought probe was “mind wandering”, then the 10 trials preceding that thought probe received a label of mind wandering, and are referred to as mind wandering trials thereafter. This time window has been previously used in ERP studies [12, 33, 37, 48] in order to maximize the number of trials that can be included to create a reliable ERP average while still maintaining a reasonable validity of the attentional report. Earlier studies have also provided detailed justification for using this time window [33, 34]. Each block ended with a thought probe. There was a total of 25 blocks, with varying block duration (i.e. 45 to 75 seconds) to prevent subjects from anticipating thought probe occurrence. Therefore, a maximum number of 250 trials (10 trials x 25 blocks) were included in subsequent analyses for each subject.

Despite the shortcomings of the thought sampling approach, it serves to provide the labelled attention reports for our supervised machine learning models (as described below). Therefore, thought sampling remains a valuable tool for validating machine learning models in mind wandering. Once sufficient evidence accumulates that validate machine learning models across different experimental paradigms, we can strive to reduce reliance on this measure in future studies.

### EEG acquisition and preprocessing

EEG was recorded reference-free continuously from 64 active electrodes mounted on a cap using the Biosemi ActiveTwo system (www.Biosemi.com). Electrodes were placed according to the International 10–20 system. Continuous EEG data were amplified and digitized at 1024 Hz, and bandpass filtered between 1Hz and 50Hz. Vertical and horizontal eye movements were recorded from electrodes above and below the right eye and two electrodes placed at the right and left outer canthus. EEG data were down-sampled to 512 Hz, then high-pass filtered at 1 Hz (as this is ideal for independent component analysis [49]), and notch filtered at 60 Hz. Electrodes with excessively noisy signals were removed and replaced with an interpolation from neighboring electrodes using spherical spline interpolation [50]. Independent component analysis was used to detect and remove eye movement and muscle artifacts. Continuous EEG data were then segmented into 3000ms epochs, beginning at 1000ms prior to stimulus onset. Each trial was visually inspected for any remaining artifacts, which were further manually removed. Common average reference was then applied to each subject’s data prior to ERP analysis. EEG data pre-processing and analysis were adapted from Kam and colleagues [51] and performed using EEGLAB [52, 53] and custom Matlab scripts.

### ERP analysis

EEG signals were bandpass filtered at 1-15Hz for ERP analysis [54–56]. All ERPs were quantified by the peak amplitude measure relative to a -200 to 0 pre-stimulus baseline. For N1, we extracted the minimum amplitude in the 80–120 ms post-stimulus time window over fronto-central midline sites (FC1, FCz, FC2), where N1 is typically maximal and also maximal in our data. For P3, we extracted the maximum amplitude in the 400–600 ms post-stimulus time window over parietal sites (P1, Pz, P2), where P3 is typically maximal and also maximal in our data.

For each ERP measure, we averaged across the channels and time points of interest as described above. We considered up to the 10 trials (excluding artifactual trials) preceding the thought probe at the end of each block, and categorized them according to the reported attention state. In order to ensure our data are consistent with previous findings, we first implemented univariate analyses to examine attentional effects in the N1 and P3 ERP components. In particular, we statistically compared the peak ERP amplitudes between on task and mind wandering states by implementing repeated measures ANOVAs for each ERP component (i.e. N1 and P3) to examine attentional effects (i.e. on task and mind wandering) while taking into account tone differences (i.e. standard and target tones). Paired samples t-tests were conducted post hoc to examine attentional effects separately for each tone. These analyses serve the purpose of ensuring that ERP patterns across on task and mind wandering states are consistent with previous studies.

For machine learning analyses, we extracted information at the block level. Specifically, we computed the mean and standard deviation across up to 10 trials for each block in order to obtain a stable ERP measure (via averaging across trials per block) and have sufficient data points per subject to input into the machine learning model (via deriving one input per block). The alternative options are suboptimal, including measuring ERPs at the single trial level which results in unreliable estimates of ERP, or averaging across all blocks to yield one grand average value per subject which results in insufficient inputs for machine learning models to classify attention states. Given the temporal fluctuations of our attention state, our approach allows us to account for the ebb and flow of attention throughout the task and its corresponding changes in the electrophysiological measures across time within individuals. Accordingly, for each block, we extracted the following features: 1) the mean across the 10 preceding trials for each ERP measure (the N1 minimum amplitude and the P3 maximum amplitude), as an index of the magnitude of electrophysiological response, and 2) the standard deviation across the 10 trials for each ERP measure, as an index of the variability in our response to external events. In essence, each subject will have up to as many inputs as blocks completed (i.e. 25) for each ERP component (i.e. N1 and P3) and descriptive measure (mean and standard deviation of ERP peak amplitudes across 10 trials per block) into the machine learning models.

### Statistical analysis

To predict attention states, we included the aforementioned ERP measures as features into a machine learning model that makes a binary classification: on task (0) or mind wandering (1). In particular, we derived the mean and standard deviation of the N1 and P3 peak amplitudes of the last 10 trials per block. For these analyses, we focused on ERP amplitudes in response to standard tones only since there were very few target tones (i.e. on average two) occurring within the last 10 trials within a block. This low number of target tones not only yields an unreliable estimate of the ERP response to target tones, but it could reduce generalization across individuals. Using Scikit-learn (version 0.20.2), we built two machine learning models to discriminate between the two attention states, including one linear model (i.e. linear logistic regression) and one non-linear model (i.e. a support vector machine (SVM) with a radial basis function (RBF) kernel). We computed the mean amplitude across all blocks in the on task state and subtracted this mean from the data for both attention states; this additional step normalizes the data, which makes the two conditions more comparable across different subjects [57]. This normalization was performed independently for each feature and each subject. We adopted this approach so that our machine learning models would likely be less impacted by individual differences in the absolute values of each EEG measure.

#### Class imbalance

The number of data points in the two attention state conditions was slightly imbalanced (*n* = 156 for on task, and *n* = 193 for mind wandering). Class imbalance often poses a challenge for supervised classifiers due to more exposure to the majority class in the training data. Similar to many other eye gaze-based mind wandering detectors [21, 25, 58, 59], we corrected for this imbalance prior to training the models using an oversampling technique to increase the number of data points in the minority class in the training data. Specifically, we used Synthetic Minority Over-Sampling Technique (SMOTE) [60], which creates new “synthetic” instances of on task (the minority class) to balance the classes in the training set; the model is therefore trained on equal numbers of both classes in order to better learn the patterns. SMOTE creates the synthetic on-task instances through a linear interpolation of the feature values from a series of “real” nearest-neighbor values in the data. SMOTE is a commonly used approach for mind wandering classification with machine learning, given that the classes are typically imbalanced [59, 61]. One subject was excluded from subsequent analyses because they did not report enough instances of the minority class (N < 5) to generate synthetic data points using the SMOTE technique. Although it is also possible to remove data points from the mind wandering condition with more data points to achieve a class balance, we implemented the SMOTE in order to maximize the amount of data that can be used for training in the models.

The models were used to classify attention states in two ways 1) within subjects (i.e. person-dependent models), the current gold standard in the literature using EEG features, and 2) across subjects (i.e. person-independent models). Classification within subjects attempts to detect mind wandering on an individual basis, using each subject’s own electrophysiological signatures of attention state for prediction. This approach is useful for maximizing prediction accuracy for that particular individual, but the model does not generalize well to new individuals. In contrast, classification across subjects attempts to accurately classify attention states in “never-before seen” subjects for greater generalizability. These models attempt to find an optimal algorithm that can predict attentional states of individuals who were not part of the training group data. This method has been successfully implemented using behavioral and pupillometry measures [19, 25]; however, no studies to our knowledge have successfully classified attention states using a generalizable model across subjects with EEG features.

#### Cross-validation methods

To classify on task vs. mind wandering *within* a subject (i.e. person-dependent models), we used k-fold cross validation, with 5 folds on 25 instances (which represent the 25 blocks of data) for each subject. In order to ensure class balance within subjects for the classification analysis, SMOTE was performed on the training set within each iteration.

For training the models *across* subjects (i.e. person-independent models) we used leave-one-subject-out cross-validation, which is similar to k-fold cross validation, with the exception that the training and testing sets are completely independent. Using this technique, one subject was reserved as the testing set, and the remaining k-1 subjects were used as the training set. This was repeated k times, where k = the number of subjects, such that each subject was used as the testing set once. This validation was performed to ensure that the training and testing sets are both exclusive and independent, and that the model generalizes across new subjects. We chose this validation technique, as opposed to the traditional k-fold cross validation, due to our limited sample size. Importantly, it closely emulates real-life applications of mind wandering detection, where a model can be previously trained on a set of data gathered from multiple subjects, and then tested on a new individual.

#### Model evaluation

Model performance was evaluated using three common metrics: accuracy, area under the curve (AUC), and Matthews Correlation Coefficient (MCC). Accuracy is the number of correct predictions of attention state over the number of total predictions, and ranges from 0 (no predictions correct) to 1 (all predictions correct). Notably, accuracy by itself is somewhat problematic when class imbalance exists, since prediction of the majority class will be inherently above 50% by chance. We therefore focus on the other two metrics that are not sensitive to class imbalance.

AUC is one of the most widely used metrics for binary classification. It is equivalent to the probability that the model will rank a randomly chosen positive example (e.g. mind wandering) higher than a randomly chosen negative example (e.g. on task). This measure can be represented as the area under the curve of a plot of the false positive rate vs. the true positive rate. AUC ranges from 0 (no predictions correct) to 1 (all predictions correct), with chance level at 0.5. MCC takes into account true and false positives and negatives, and outputs a value that ranges from -1 (no predictions correct) to 1 (all predictions correct), with chance level at 0. Together, these metrics demonstrate whether the models are capable of successful classification of attention states, and assess classification performance with respect to chance and imbalanced classes.

We additionally evaluated confusion matrices of the best performing models, which allowed us to visualize the performance of each model and summarize the true and false positives and negatives. The confusion matrix reveals what type of errors the model makes: specifically, whether the model shows any bias or skew towards a particular class, and if so, how. It is possible to examine the true positive rate (sensitivity) and true negative rate (specificity) to determine which of the two models result in more accurate prediction. Similarly, it is possible to examine the false positive and false negative rates to determine if the model leans towards any decision-making errors. For example, a high sensitivity and high false positive rate may point to over-classification of the positive instance.

## Results

### Behavioral performance

**Fig 1:**
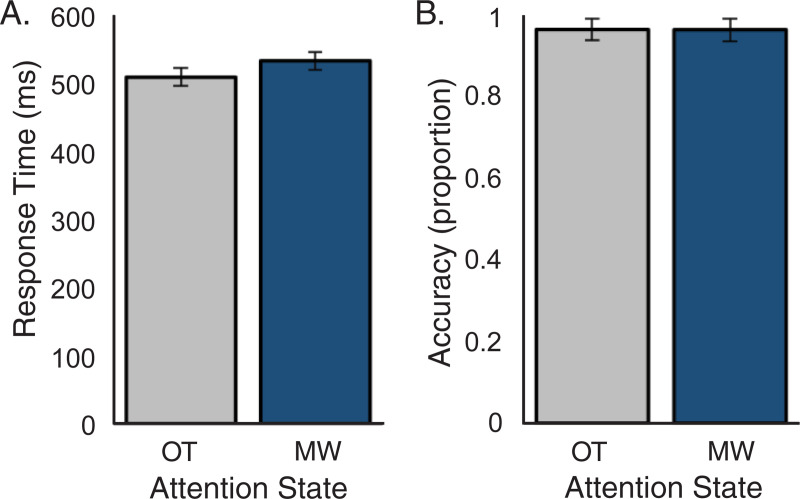
Behavioral results as a function of attention state. (A) Mean response time was slower during mind wandering (p = 0.076). (B) No difference was observed in accuracy between the two attention states. Error bars = standard error of the mean; OT = on task; MW = mind wandering.

Subjects reported mind wandering 55% of the time (range: 20%– 88%, *SE* = 5.3%) and being on task 45% of the time. This is consistent with the typical breakdown of self-reported attention states in the literature for this type of task [34, 35, 37]. Mean reaction time and accuracy are shown in Fig 1. There was a trend for slower response time during mind wandering (*M* = 535 ms, *SE* = 14 ms) compared to on task periods (*M* = 511 ms, *SE* = 14 ms; *t*(12) = -1.94, *p* = 0.076). However, accuracy did not differ between attention states for mind wandering (*M* = 0.9619, *SE* = 0.030) compared to on task (*M* = 0.9623, *SE* = 0.028; *t*(12) = 0.098, *p* = 0.924).

### Univariate analyses on ERP components

In order to check the veracity of the attentional reports, we first evaluated whether our ERP measures reveal attentional differences that are consistent with previous studies. Specifically, if our ERP measures show reduced amplitudes during mind wandering, this would provide corroborating evidence for the attentional reports. To assess conditional differences in ERP measures, we implemented repeated measures ANOVA with attention (on task and mind wandering) and tone (standard and target) as within subject factors, separately for the N1 and P3 ERP components. These results are reported in Table 1.

**Table 1:** ANOVAs on ERP components.

Features	Attention (OT vs. MW)	Tone (Standard vs. Target)	Attention x Tone Interaction
N1 Min	*F*(1,52) = 3.57, *p* = 0.064	*F*(1,52) = 6.23, *p* = 0.013	*F*(1,52) = 1.51, *p* = 0.224
P3 Max	*F*(1,52) = 1.79, *p* = 0.187	*F*(1,52) = 58.99, *p* < .001	*F*(1,52) = 1.24, *p* = 0.271

*Note*: MW = mind wandering. OT = on task.

Repeated measures ANOVAs of main effects of attention and tone as well as attention × tone interaction, reported separately for N1 and P3 ERP components.

**Fig 2:**
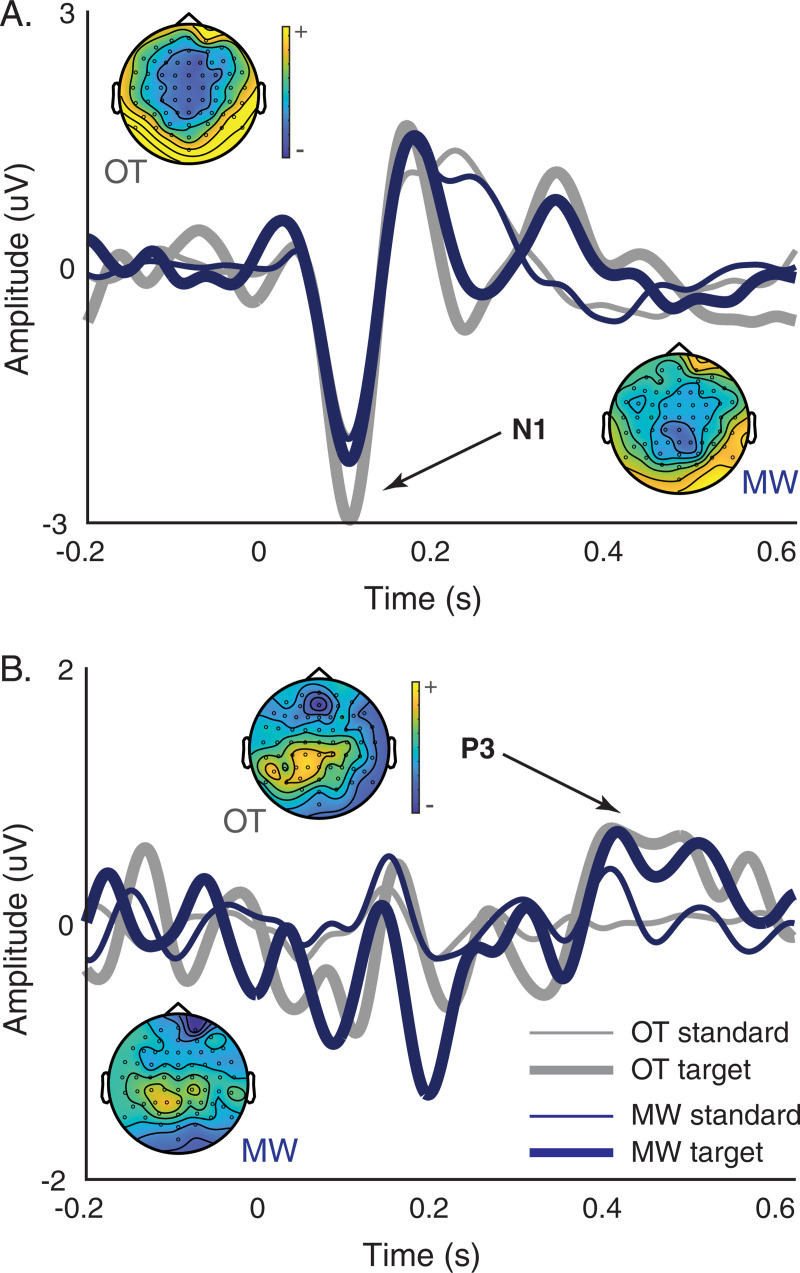
Grand average ERP waveforms. N1 was averaged across FC1, FCz and FC2 (left panel), whereas P3 was averaged across P1, Pz and P2 (right panel). Univariate analyses indicate reduced N1 in response to target tones during MW relative to OT periods. OT = on task, MW = mind wandering.

As expected, the main effect of tone was significant for both the N1 and P3, with increased amplitude during target tones relative to standard tones. For N1, there was a near significant main attentional effect, characterized by reduced N1 during mind wandering relative to on task periods. Although the attention x tone interaction was not significant, we implemented follow up analyses using paired samples t-tests to examine whether attentional effects varied for each tone as was observed in Fig 2. Consistent with previous findings of attentional effects in sensory ERP components [33, 36], our analyses revealed a significant attentional effect for the N1 in response to target tones (*t*(13) = -2.83, *p* = 0.014) but not standard tones (*t*(13) = -0.72, *p =* 0.485). Neither the main attention effect nor interaction effect were significant for the P3.

### Machine learning

#### Person-dependent classification performance

Individual accuracy for subjects with the non-linear SVM with RBF kernel ranged from 0.383 to 0.960 (*M* = 0.643, *SE* = 0.044), whereas accuracy for the linear logistic regression model ranged from 0.375 to 0.843 (*M* = 0.621, *SE* = 0.038). The range of accuracy values observed in our study, as well as the variability in accuracy rates across individuals, are similar to accuracy values reported in prior work using EEG measures for predicting mind wandering [44, 46]. Although accuracy might be useful for comparing findings across different studies, this metric can be biased with class imbalance (as previously mentioned); therefore, it is more informative to examine the AUC and MCC values for individual subjects.

**Fig 3:**
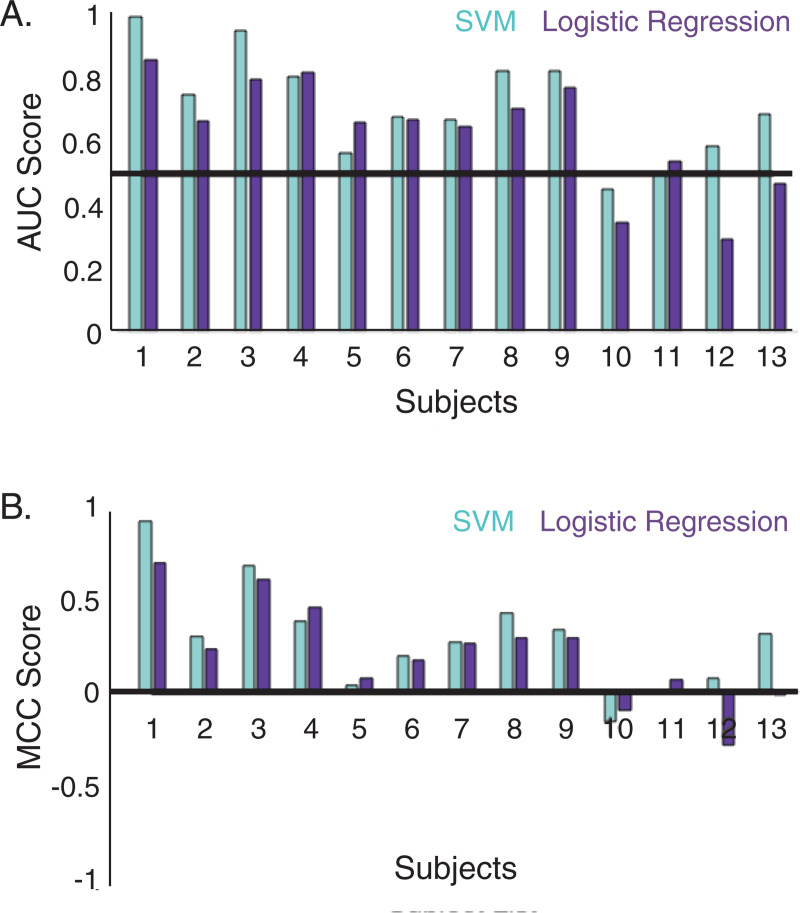
Model performance per subject, as measured by AUC and MCC. AUC performance for each subject for both models (SVM and logistic regression) is shown in top panel; chance is noted by the black horizontal line at 0.5. MCC performance for each subject is shown in bottom panel; chance is noted by the black horizontal line at 0. AUC = area under the curve; MCC = Matthews correlation coefficient; SVM = support vector machine.

Both AUC and MCC metrics also demonstrate, on average, above-chance performance for the SVM and logistic regression models. AUC ranged from 0.452 to 0.995 (*M* = 0.715, *SE* = 0.0457) for the SVM and 0.291 to 0.858 (*M* = 0.635, *SE* = 0.0491) for the logistic regression model. MCC ranged from -0.162 to 0.919 (*M* = 0.289, *SE* = 0.0792) for the SVM and -0.287 to 0.694 (*M* = 0.210, *SE* = 0.0758) for the logistic regression model. Machine learning performance as indexed by AUC and MCC for each individual subject is shown in Fig 3.

#### Person-independent classification performance

In this method of classification, we aimed to generalize the model across subjects by using leave-one-subject-out cross-validation. Both the nonlinear SVM and logistic regression models performed above chance (MCC > 0, AUC > 0.5). Accuracy ranged from 0.514 to 0.750 (*M* = 0.591, *SD* = 0.070) for the SVM, and from 0.461 to 0.752 (*M* = 0.588, *SD =* 0.093) for the logistic regression model. MCC values ranged from –0.026 to 0.446 (*M* = 0.206, *SD* = 0.152) for the SVM and –0.056 to 0.449 (*M* = 0.196, *SD* = 0.169) for the logistic regression. Finally, AUC values ranged from 0.485 to 0.731 (*M* = 0.613, *SD* = 0.085) for the SVM and 0.468 to 0.739 (*M* = 0.609, *SD* = 0.096) for the logistic regression model. Overall, the nonlinear SVM showed the best performance, as indicated by higher scores in accuracy, AUC, and MCC, as compared to logistic regression. Although there is clear variability in the evaluation metrics across participants (as is common for other real-time detectors [46]), we suggest the average model performance faired quite well for most individuals. A summary of the results for each model are reported in Table 2.

**Table 2:** Model evaluation of person-independent classification performance.

Models	Performance Metrics		
	Accuracy	AUC	MCC
SVM with RBF Kernel	0.591 (SD = 0.070)	0.613 (SD = 0.085)	0.206 (SD = 0.152)
Logistic Regression	0.588 (SD = 0.093)	0.609 (SD = 0.096)	0.196 (SD = 0.169)

*Note*: Classification performance indices, including accuracy, AUC, and MCC, are reported for both machine learning models: SVM with RBF kernel and logistic regression. AUC = area under the curve; MCC = Matthews correlation coefficient; SVM = support vector machine; RBF = radial basis function.

The ultimate goal of a mind wandering detector is to assess the phenomenon in real time for measurement and interventions; thus precision (i.e. accurately classifying instances of mind wandering as mind wandering) is a priority of the model. We therefore evaluated the confusion matrices of the best models to determine the type of errors the models made (as shown in Table 3). Neither model exhibited any skew towards sensitivity (i.e. true positives for predicting mind wandering) or specificity (i.e. true negatives or correct rejections). Taken together, the SVM model outperformed the logistic regression model. Not only did the SVM model show higher sensitivity and specificity, it also showed higher values for AUC and MCC, which are metrics that are robust against the presence of imbalanced classes.

**Table 3:** Confusion matrices of person-independent models.

		Actual MW	Actual Not MW
**SVM with RBF Kernel**	Pred. MW	0.570	0.390
	Pred. Not MW	0.430	0.610
**Logistic Regression**	Pred. MW	0.528	0.356
	Pred. Not MW	0.472	0.644

*Note*: Confusion matrix for each of the machine learning models: SVM with RBF kernel and logistic regression. Pred. = predicted; MW = mind wandering; SVM = support vector machine; RBF = radial basis function

#### Feature analysis

To further examine each feature individually, we created models that were trained on only one feature at a time (Table 4). Given that SVM outperformed logistic regression, we implemented these analyses with the SVM model only. This post-hoc analysis allowed us to identify the feature that is most effective in predicting attention states. Our results indicate that most features performed slightly above chance, with the peak P3 amplitude showing the best performance. Notably, none of the features individually performed as well as the complete model.

**Table 4:** Model performance for individual features.

Features	Performance Metrics		
	Accuracy	AUC	MCC
**N1 Min**	0.489	0.521	0.033
**N1 SD**	0.322	0.471	-0.084
**P3 Max**	0.562	0.594	0.171
**P3 SD**	0.518	0.567	0.106

*Note*: Model performance metrics, including accuracy, AUC, and MCC, implemented separately for each individual feature of ERP components. Models for all four features were built with the SVM with RBF kernel. SD = standard deviation. AUC = area under the curve; MCC = Matthews correlation coefficient; SVM = support vector machine; RBF = radial basis function.

## Discussion

Mind wandering is an intrinsically covert state that is consistently linked to negative affect and performance decrements. Although it is traditionally measured via self-reports, our proof-of-concept study attempts to overcome the shortcomings of this approach by developing machine learning models that can reliably predict its occurrence using EEG measures that directly and objectively capture neural activity. Such models will be crucial to the development of applications that can mitigate the negative effects of mind wandering in real-time [18], as well as future research that will no longer need to rely on constant task interruptions to determine when mind wandering occurs using thought sampling.

During a target detection task, our univariate analyses demonstrated significant attentional differences in the sensory ERP components. Our machine learning classifiers using both the support vector machine and logistic regression models were able to classify mind wandering at above-chance levels within subjects. Notably, we improved on prior work in this area by developing person-independent models that can detect mind wandering using EEG features without having any prior information about that person. The performance of our person-independent models is modest, but comparable to previous studies showing generalizability across subjects using behavioral measures, eye gaze, and pupillometry [25, 58, 61]. These findings underscore, for the first time, the potential for generalizable machine learning models that can classify mind-wandering states in real-time using electrophysiological measures.

Using machine learning methods with EEG measures to predict mind wandering, we had better success with the SVM model relative to the logistic regression model in both within- and across-subject classification of mind wandering states. This finding is consistent with previous EEG studies that demonstrate the effectiveness of nonlinear models in determining the boundary between attention states [43, 44, 46], highlighting the utility of nonlinear models in classification accuracy and generalizability. Examination of the confusion matrix revealed that the models were not biased in their predictions, with both models showing negligible difference between sensitivity and specificity. These unbiased results may be due to the oversampling technique, SMOTE, which addressed the issue of class imbalance between attention states. The effectiveness of SMOTE has been demonstrated in other work using behavioral measures in detecting mind wandering [21, 59]. A previous study that classified mind wandering states using EEG has reported issues with highly disparate sensitivity and specificity using other techniques to balance class sizes [44]. These disparities could be attributed to either the method used to achieve balanced classes (such as SMOTE) or the accuracy of subjects’ responses during thought sampling. Although this is a well-validated approach in measuring one’s attentional state [2, 62], thought sampling does nevertheless rely on self-reports and therefore depend on accurate and honest responses from subjects. Any bias in subjects’ responses could reduce classification accuracy and potentially result in the observed difference between sensitivity and specificity. This limitation may explain parts of our data in which model performance was below chance for at least one subject in the person-dependent model, suggesting that machine learning models are effective in general in predicting attention states but not necessarily for every single individual.

Finally, we examined each feature individually by creating separate SVM models for each feature, in order to evaluate its relative importance in classification accuracy. Interestingly, the peak P3 amplitude resulted in the highest accuracy, AUC, and MCC, despite not showing significant attentional differences in the univariate analyses. These contrasting results may be puzzling at first glance; however, the differing nature of the averaged ERP amplitudes entered into the univariate analyses and machine learning models may potentially account for this difference. While the univariate analyses involved the peak amplitudes averaged across the entire task, the machine learning models received the peak amplitudes averaged within each of the 25 blocks as input; therefore, it takes into account fluctuations in the P3 amplitude throughout the task. That peak P3 amplitude contributed to classification accuracy but did not show attentional differences in univariate analyses suggests that statistical significance at the univariate level captures different information compared to machine learning classifiers. Future work can clarify the relationship between these types of input data and their corresponding analyses. These findings underscore the value of considering multivariate patterns of data via machine learning models in classifying temporally fluctuating attentional states.

Importantly, machine learning analyses take into consideration the multivariate aspects of EEG data, and thereby complement traditional univariate analyses that focus on individual features. Our univariate analyses revealed attentional differences at the sensory level but not higher cognitive level ERP component. Although the peak N1 amplitude in response to target tones was greater during on task compared to mind wandering states [33, 36], we did not observe any attentional differences in the N1 and P3 in response to standard tones. In contrast, our machine learning analyses using ERP components in response to standard tones were successful at detecting mind wandering based on these features. These findings highlight the value of considering multiple features of EEG data in predicting attentional states.

Future work may involve efforts to improve model performance by adding additional data and more features. Effective machine learning models generally rely on large amounts of data. Although our proof-of-concept study showed that even with a small sample size, electrophysiological markers can predict mind wandering within subjects and across subjects, these results need to be replicated in future studies with a larger sample size. Notably, a larger sample size will provide more training data, and thus it can only enhance accuracy further in the across subjects analyses. Our finding makes a methodological contribution, namely that accurate machine learning models of mind wandering can be derived from a sample size of 14 participants, providing the lower threshold in sample size needed to predict mind wandering above chance across subjects.

Increasing the number of data points is one way of improving prediction accuracy; another way is to include multi-modal sources of data. Combining EEG measures with behavioral and pupillometry features could result in models with better performance, since both types of measures have resulted in moderately successful models in previous studies [19, 21, 25]. Other promising avenues that could improve prediction rates include feature crossing and ensemble learning, which are techniques that have yet to be implemented often in prior research nor this current study. The former entails creating synthetic data by multiplying or crossing two or more features, which may provide predictive abilities beyond those features individually. The latter is a technique that combines several different machine learning models to minimize causes of error and improve performance. Another future direction could involve identifying EEG markers of the types of thoughts we engage in during mind wandering, such as autobiographical memory retrieval or future planning. Results in this present study demonstrate that SVM and logistic regression classifiers can detect mind wandering; therefore, these models can potentially be used to further classify task-unrelated thought as a function of its temporal focus.

In summary, this study provides evidence that electrophysiological markers can be employed in machine learning models to detect mind wandering. Our SVM and logistic regression classifiers were capable of generalizing across individuals, which has not yet been demonstrated in other studies that utilize EEG markers. Moreover, our models performed at above chance levels as determined by several metrics, which is especially promising given that univariate analyses of the same features did not always show attentional differences. Taken together, this research brings us closer to the possibility of more intelligent programs that could detect mind wandering in real-life situations, in real-time.
